# RARγ-induced E-cadherin downregulation promotes hepatocellular carcinoma invasion and metastasis

**DOI:** 10.1186/s13046-016-0441-9

**Published:** 2016-10-19

**Authors:** Wen-Juan Gan, Jing-Ru Wang, Xiao-Li Zhu, Xiao-Shun He, Peng-Da Guo, Shen Zhang, Xiu-Ming Li, Jian-Ming Li, Hua Wu

**Affiliations:** 1 Pathology Center and Department of Pathology, Soochow University, Suzhou, 215123 China; 2 The First Affiliated Hospital of Soochow University, Suzhou, 215006 China

**Keywords:** RARγ, E-cadherin, Hepatocellular carcinoma, Metastasis

## Abstract

**Background:**

Aberrant expression of Retinoic acid receptor γ (RARγ) is implicated in cancer development. Our previous study identified that RARγ functions as a tumor promoter to drive hepatocellular carcinoma (HCC) growth. However, its contribution to HCC invasion and metastasis remains unclear.

**Methods:**

RARγ expression in clinical HCC samples was detected by western blot and immunohistochemistry. The relationship between RARγ expression levels and the clinical characteristics were evaluated. HCC cell line MHCC-97H were stably knocked down RARγ using a lentivirus vector-based shRNA technique. The cells were analyzed by migration and invasion assays, and injected into nude mice to assess tumor metastasis. E-cadherin expression regulated by RARγ was examined by qPCR, western blot and immunofluorescence staining.

**Results:**

The expression of RARγ is significantly upregulated in human HCC tissues. Moreover, its expression positively correlates with tumor size, distant metastasis and TNM stage, and negatively correlates with length of survival of HCC patients. Knockdown of RARγ markedly inhibits HCC cell invasion and metastasis both in vitro and in vivo. Mechanistic investigations reveal that RARγ functions through regulation of NF-κB-mediated E-cadherin downregulation to promote HCC invasion and metastasis. Notably, RARγ expression status negatively correlates with E-cadherin expression in HCC cell lines and clinical HCC samples.

**Conclusions:**

These findings demonstrate that RARγ could promote HCC invasion and metastasis by regulating E-cadherin reduction, and implicate new strategies to aggressively treat HCC through targeting RARγ/E-cadherin signaling axis.

**Electronic supplementary material:**

The online version of this article (doi:10.1186/s13046-016-0441-9) contains supplementary material, which is available to authorized users.

## Background

Hepatocellular carcinoma (HCC) is one of the most common cancer types worldwide, particularly in China [1, 2]. HCCs that undergo early vascular invasion are highly resistant to existing therapies, such as chemotherapy [3]. Although current advances have been made in the diagnosis of HCC, the overall survival rate of the HCC patients is disappointingly low due to recurrence and metastasis [4, 5]. Extensive studies have identified that many risk factors such as hepatitis B (HBV) and hepatitis C (HCV), and dysregulation of signaling pathway and molecules, are implicated in HCC development [6, 7]. However, little is known about how HCC undergoes metastasis. Therefore, identifying potential molecular mechanisms contributing to the metastasis of HCC is one of the most critical issues.

Metastasis, a complex biological process that involves tumor cells detaching from the primary tumor, migrating and locating in distant organs, is the principal cause leading to death in all types of cancer including HCC [8, 9]. Significant advances have been made in understanding the molecular mechanisms underlying metastasis, and several signalings and molecules have been identified [10]. Accumulating evidence indicates that epithelial-mesenchymal transition (EMT) is a crucial step for cancer cell invasion and metastasis initiation [11]. Downregulation of E-cadherin, a single-span transmembrane glycoprotein located primarily within adherent junction, is a fundamental feature of EMT [8, 12–14]. Loss of E-cadherin correlates with poorer survival for patients with numerous cancers such as gastric cancer and HCC [15, 16]. Loss or reduction of E-cadherin expression in human tumors can be caused by somatic mutations, chromosomal deletions, DNA methylation and several intracellular EMT-inducers like Twist, Snail, Slug, Zeb1, Zeb2 and others [17–26]. For example, somatic point mutations of the E-cadherin gene [17] and/or promoter methylation lead to E-cadherin loss [20, 27], and the loss of E-cadherin expression is considered to be a distinguishing feature in both lobular breast cancer and diffuse gastric cancer [27, 28]. Transcriptional repressors, such as Snail, Slug and Twist, directly bind to the E-cadherin promoter to transcriptionally repress E-cadherin expression in breast cancer [22, 29]. We recently also reported that Nur77, an orphan member of the nuclear receptor superfamily, induces E-cadherin reduction in a Matrix metalloproteinase 9 (MMP-9)-dependent manner, and subsequently contributes to the invasion and metastasis of colorectal cancer [8]. Despite these efforts to understand the molecular mechanism of E-cadherin ablation or reduction in cancer, however, regulation of E-cadherin expression in cancer is still poorly understood.

Retinoic acid receptor γ (RARγ) is a member of the nuclear receptor subfamily. Recent studies implicate RARγ in cancer development and progression. A lot of efforts have been made to explore the regulatory mechanism of RARγ in cancer, but there are contradictory views regarding its role in cancer. On one hand, RARγ may function as an oncogene to drive cancer cell growth and metastasis. For example, RARγ upregulation in cholangiocarcinoma contributes to cancer cell growth and metastasis [30]. We previously also reported that RARγ is overexpressed in HCC and overexpression of RARγ endows HCC cells with malignancy types [31]. In contrast, RARγ has also been reported as a tumor suppression factor that inhibits cancer cells proliferation and invasion. For example, RARγ, by regulating the expression of carbohydrate sulfotransferase 10 (CHST10), can suppress melanoma invasion [32]. Loss of RARγ, but not RARα, promotes v-Ha-Ras-induced squamous cell carcinoma [33]. Recently, we reported that RARγ is downregulated in clinical colorectal cancer tissues, and RARγ acts as a tumor suppressor to regulate colorectal tumorigenesis and metastasis by restricting the Hippo-Yap pathway-mediated oncogenic signaling [34]. Taken together, these results strongly indicated that RARγ plays a pivotal role in cancer development. However, it is unknown whether and how RARγ is involved in HCC invasion and metastasis.

In the present study, we investigated the role of RARγ in the invasion and metastasis of HCC. Our in vitro and in vivo studies demonstrate that RARγ, acting through NF-κB-mediated E-cadherin reduction, drives HCC cell invasion and metastasis. In clinical HCC samples, we observed a statistical correlation between elevated RARγ expression and distant metastasis and poor survival. These findings implicate that nuclear receptor RARγ may be used as a new potential target for aggressive HCC therapy.

## Methods

### Antibody and regents

Anti-RARγ, anti-E-cadherin and anti-Myc tag antibodies were purchased from Santa Cruz Biotechnology (Santa Cruz, CA, USA). all-*trans*-RA (ATRA) and anti-β-actin were purchased from Sigma-Aldrich (St. Louis, MO, USA). Lipofectamine 2000 and TRIZOL reagent were purchased from Invitrogen (Carlsbad, CA, USA) and WesternBright ECL reagents were purchased from Advansta (Menlo Park, CA, USA).

### Cell culture

The QGY-7703, MHCC-97H, SMMC-7721, BEL-7402, SK-HEP-1 and Huh-7 human hepatocellular carcinoma cell lines were purchased from the Cell Bank of the Chinese Academy of Sciences (Shanghai, China). These cells were cultured in DMEM medium containing 10 % fetal bovine serum at 37 °C in a humidified 5 % CO2 atmosphere.

### Tissue samples and evaluation

Fifty-six human HCC tissues were obtained from the First Affiliated Hospital of Soochow University (Suzhou, Jiangsu, China) from 2012 to 2013. Expression levels of RARγ and E-cadherin were scored using immunohistochemistry in all tissues. The clinical characteristics of all patients included in this study are listed in Table 1. The correlation of RARγ expression and patients' survival outcomes were illustrated using a HCC tissue microarray (Outdo Biotech Co., Ltd, Shanghai, China) containing 90 HCC samples with survival time. These samples were collected between 2002 and 2009. The study was approved by Soochow University for Biomedical Research Ethics Committee, and all of the patients provided informed consent. The staining score was evaluated as recently described [35, 36].

**Table 1:** Correlation of RAR$\gamma$ expression with patients’ clinicopathological variables in 56 cases of HCCC

Characteristics	All cases (*N* = 56)	RAR$\gamma$ expression (%)			
		Low (*n* = 21)	High (*n* = 35)	$\chi$ ^2^ value	*p* value
Gender				0.221	0.639
Male	47	17	30		
Female	9	4	5		
Age (years)				1.026	0.311
≤ 50	15	4	11		
> 50	41	17	24		
Tumor size (cm)				5.265	0.022
≤ 3	8	5	3		
> 3	48	11	37		
Cirrhosis				0.076	0.783
Negative	28	10	18		
Positive	28	11	17		
HBV infection				0.327	0.567
Negative		4	9		
Positive		17	26		
Distant metastasis				8.443	0.004
No	26	15	11		
Yes	30	6	24		
TNM stage				8.310	0.004
I/II	41	20	21		
III/IV	15	1	14		
Differentiation				0.448	0.799
Well	6	3	3		
Moderate	25	9	16		
Poor	25	9	16		

### Generation of stable cell lines

MHCC-97H cell lines stably expressing RARγ-specific shRNA (shRNA/RARγ) or scrambled shRNA control (shRNA/Control) were constructed using a lentiviral shRNA technique (GeneChem, Shanghai, China). The detailed protocol has been described recently [8]. The human RARγ shRNA target sequences were as follows: shRNA/RARγ 1#, 5'-CTCCCTTAATCCGAGAGAT-3'; and shRNA/RARγ 2#, 5'-CTCAGTTAGAAGAGCTCAT-3'.

### Western blot and immunofluorescence stainng

Western blot was performed as described recently [8]. Protein expression was detected using primary and secondary antibodies, and visualized using enhanced chemiluminescence reagents and autoradiography. Representative blots are shown from three independent experiments. Cells for immunofluorescence staining were grown and stained as previously described [37]. The images were taken with a Nikon ECLIPSE Ni scope with color camera and were processed by NIS-Elements D 4.10.00 software.

### RNA extraction and qPCR analysis

Total RNAs were extracted and reverse transcribed as recently described [8, 38]. qPCR was undertaken using gene-specific primers for E-cadherin with Power SYBR® Green PCR Master Mix (TaKaRa, Japan). Normalization was performed with β-actin. The following primers were used: E-cadherin, forward 5'-GTCACTGACACCAACGATAATCCT-3' and reverse 5'-TTTCAGTGTGGTGATTACGACGTTA-3'; β-actin forward 5'-CACCAACTGGGACGACATG-3' and reverse 5'-GCACAGCCTGGATAGCAAC-3'.

### Transwell migration and Matrigel invasion assays

For transwell migration assays, 7.5 × 10^4^ MHCC-97H cells in 0.5 ml serum-free DMEM medium were added to the top chamber, and the bottom chamber was filled with 0.5 ml DMEM medium with 10 % FBS. After 24 h, cells located on the upper surface were removed using a cotton swab, and the cells on the lower surface were fixed with 100 % methanol for 5 min, and then stained with Wright-Giemsa at room temperature. For matrigel invasion assays, BD BioCoat Matrigel Invasion Chambers (Catalog No. 354480) were used for the invasion assay according to the instructions of the manufacturer. To quantify the migratory and invasive cells microscopically, cells were counted in five random fields (magnification, 200×).

### Metastasis of Xenografts

In this study, 1 × 10^6^ MHCC-97H/shRNA/Control or MHCC-97H/shRNA/RARγ cells were injected into the lateral vein in the nude mouse tail (BALB/c, SPF grade, 4–5 weeks, male). After 7 weeks, mice of each group were killed. Lung tissues were collected for metastatic foci evaluation and standard histopathological study. All animal experiments were approved by the Animal Care and Use Committee of Soochow University.

### Statistical analysis

Each assay was performed in three independent experiments. Data were presented as mean ± s.d. Statistical significance was analyzed using Student's *t*-test (unpaired, two-tailed) or one-way ANOVA. The relationships between RARγ expression and clinicopathological factors were analyzed using Pearson’s chi-square test, and the correlations between the expression levels of RARγ and E-cadherin were calculated using Spearman's rank Correlation analysis. The Kaplan-Meier survival analysis was used to illustrate the prognostic relevance of RARγ in univariate analysis. *p* < 0.05 was considered statistically significant.

## Results

### RARγ is elevated in HCC specimens and correlates with distant metastasis and poor survival

**Fig. 1:**
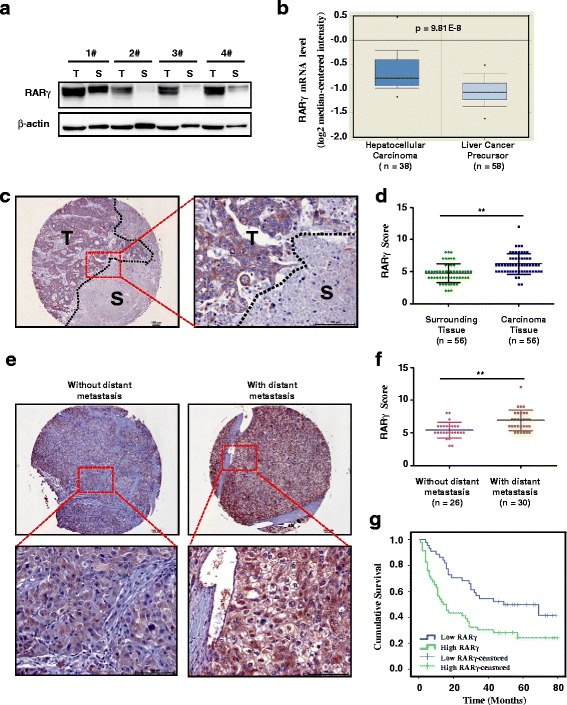
Increased levels of RARγ in HCC correlates with distant metastasis and predicts poor clinical outcome. **a** RARγ expression in HCC samples was evaluated by western blotting. Four randomly selected pairs of HCC tumors (T) and matched surrounding tissues (S) are presented. **b** Box plot shows the mRNA levels of RARγ expression in 38 human HCC and 58 human liver cancer precursor tissues. Statistical significance was determined by a two-tailed, unpaired Student's t-test. **c** Representative bright-field images showing RARγ staining (brown) in human HCC sections. Nuclei (blue) were marked by hematoxylin staining. Scale bar: 100 μm. T, tumor. S, surrounding tissue. **d** Scatter plot analysis of RARγ levels in 56 HCC tissue samples and their surrounding tissues. Statistical significance was determined by a two-tailed, paired Student's t-test. **p < 0.01. **e** Representative bright-field images showing RARγ staining (brown) in human HCC tissues with distant metastasis (*N* = 30) and without distant metastasis (*N* = 26). Nuclei (blue) were marked by hematoxylin staining. Scale bar: 100 μm. **f** Scatter plot analysis of RARγ levels in human HCC tissues with distant metastasis (*N* = 30) and without distant metastasis (*N* = 26). Statistical significance was determined by a two-tailed, unpaired Student's t-test. **p < 0.01. **g** Kaplan-Meier survival curve of HCC patients with low (*n* = 44) and high (*n* = 46) RARγ expression

To determine the role of RARγ in HCC invasion and metastasis, western blotting was first performed to examine the expression of RARγ in human HCC tissues. Compared with the matched surrounding tissues of HCC, an overexpression of RARγ was detected in the primary HCC tumors (Fig. 1a). Furthermore, we examined gene expression data from Oncomine, and found that RARγ mRNA levels are significantly upregulated in HCC tissues compared with liver cancer precursor tissues (Fig. 1b). In agreement with these results, by analyzing an additional 56 cases HCC samples, we found that RARγ expression is overexpressed in tumor tissues (Fig. 1c and d), and further upregulated in those with lymph node metastasis (LNM) (Fig. 1e and f), suggesting RARγ’s potential role in invasive progression of HCC.

We further analyzed the relationship between the levels of RARγ expression and the clinicopathological status of patients with HCC. As shown in Table 1, patients with higher RARγ expression are significantly associated with larger tumor size, distant metastasis and high TNM stage of HCC. However, there is no significant correlation between RARγ expression and other clinicopathological features, such as patient gender, age, cirrhosis, HBV infection and differentiation.

We next sought to determine whether RARγ expression in HCC is associated with patient survival time. Kaplan-Meier analysis revealed that patients with high RARγ expression have poorer overall survival. The median survival time of HCC patients with high RARγ expression was < 20 months, while the median survival time was significantly longer (~70 months) in those with low RARγ expression (Fig. 1g). These data suggest that elevated RARγ expression may contribute to HCC progression and its expression may be a valuable predicting factor for survival in HCC patients.

### Knockdown of RARγ expression inhibits HCC cell migration and invasion in vitro, and metastasis in vivo

**Fig. 2:**
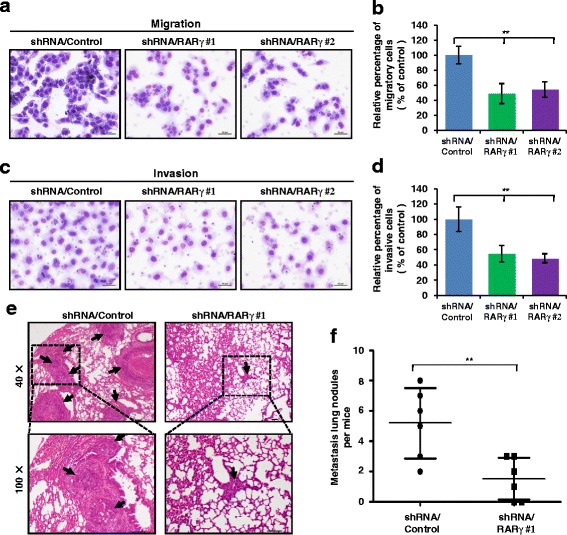
Silencing RARγ impaires HCC migration and invasion in vitro and metastasis in vivo. **a**–**d** Migration (a) and invasion (c) assays were performed in wild-type MHCC-97H cells (shRNA/Control) and in the MHCC-97H cells with stable knock down of RARγ (shRNA/ RARγ), and the relative number of migratory (b) and invasive (d) cells were calculated with Wright-Giemsa staining. **e** Knockdown of RARγ inhibits HCC metastasis. Representative lung tissue sections from each group were shown by hematoxylin and eosin staining (magnification: × 40). Black arrows indicate lung tissues with matastatic nodules. **f** The number of lung metastatic foci in each group (*n* = 6 per group) was counted under the microscope. Statistical significance was determined by a two-tailed, unpaired Student's t-test. **p < 0.01

The above findings indicate the involvement of elevated RARγ expression in HCC aggressiveness. Based on these findings, we investigated whether RARγ plays a critical role in regulating HCC cell invasion and metastasis. We first used a lentiviral-mediated shRNA technique to stably knock down RARγ expression in MHCC-97H cell lines that express a high level of RARγ protein. The knockdown efficiency was confirmed by western blotting (Additional file 1: Figure S1). Migration assays show that knockdown of RARγ largely impairs the ability of HCC cell migration, as compared with control cells (Fig. 2a and b). Similarly, invasion assays also reveal that knockdown of RARγ potently inhibits HCC cell invasive properties (Fig. 2c and d). We further examined the effect of RARγ on HCC metastasis by establishing mouse model for studying HCC metastasis in vivo. MHCC-97H cells having high metastatic potential were used for the study. Our results show that depletion of RARγ greatly impairs HCC metastasis to the lungs, indicated by the fact that MHCC-97H/shRNA/Control cells form more and larger pulmonary micrometastases than the MHCC-97H/shRNA/RARγ cells (Fig. 2e). These findings are also summarized in Fig. 2f. Evidently, these results indicate that RARγ acts to promote the invasive property of HCC cells in vitro and in vivo.

### RARγ regulates E-cadherin expression

**Fig. 3:**
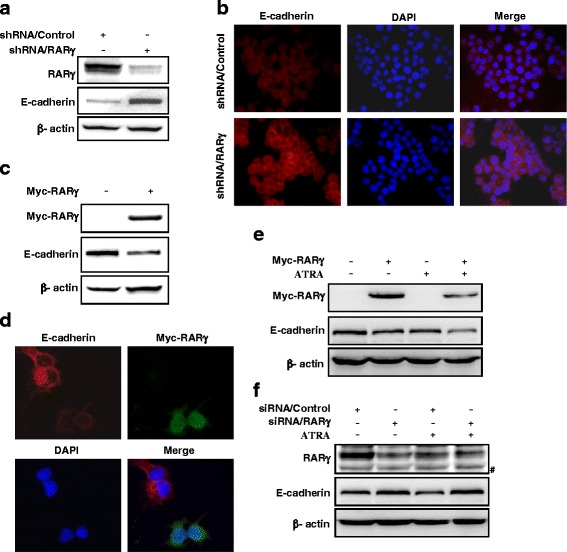
RARγ regulates E-cadherin expression. **a**, **b** silencing RARγ increases endogenous levels of E-cadherin proteins. MHCC-97H cells stably expressed shRNA/Control or shRNA/RARγ, then (a) total cell lysates were subjected to immunoblotting to determine E-cadherin levels, or (b) the cells were subjected to immunofluorescent staining of E-cadherin (red). Nuclei was stained with DAPI (blue). Representative images are shown. **c**, **d** RARγ overexpression decrease endogenous E-cadherin protein levels. Huh-7 cells were transiently transfected with vector or Myc-tagged RARγ, then endogeous protein levels of E-cadherin were detected by immunoblotting (c) or immunofluorescent staining (d). **e**, **f** The role of ATRA in RARγ-induced E-cadherin downregulation. Immunoblotting of E-cadherin in RARγ-transfected Huh-7 cells (e) or in RARγ-silenced MHCC-97H cells (f) treated with vehicle or 1 μM ATRA. #_,_ no specific band

Given that RARγ contributes to HCC invasion and metastasis, we investigated that the effect of RARγ on EMT, a critical event in cancer cell invasion and metastasis. Interestingly, as shown in Fig. 3a, knockdown of RARγ in MHCC-97H cells significantly enhanced the expression of E-cadherin, an epithelium marker in EMT. This result was also confirmed by immunofluorescent staining (Fig. 3b). Conversely, overexpression of RARγ in Huh-7 cells that express a low level of RARγ protein markedly decreased E-cadherin expression as revealed by the western blot (Fig. 3c) and immunofluorescent staining (Fig. 3d). However, we did not observe any significant change in expression of other EMT markers such as Vimentin, N-cadherin and occludin (Additional file 2: Figure S2). We next examined whether ATRA, an agonist for RARs, has an effect on E-cadherin expression. Our results show that treatment of Huh-7 cells with ATRA enhances RARγ-induced E-cadherin reduction (Fig. 3e). However, ATRA has no significant effect on the MHCC-97H cells in which RARγ is knocked down by siRNA (Fig. 3f), suggesting that RARγ-mediated E-cadherin reduction can be regulated by its ligands.

### RARγ-induced E-cadherin reduction is dependent on NF-κB Activation

**Fig. 4:**
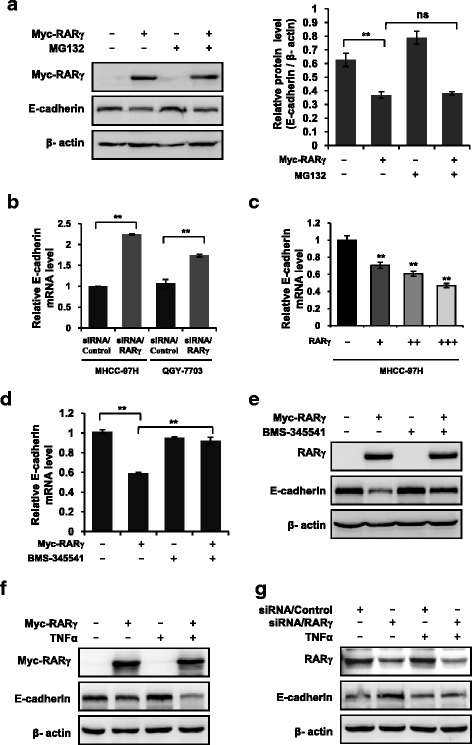
NF-κB is indispensable for RARγ-driven E-cadherin reduction. **a** RARγ-driven E-cadherin reduction does not depend on proteasome pathway. Immunoblotting (left) or qPCR (right) analysis of the E-cadherin expression in RARγ-transfected Huh-7 cells treated with vehicle or 10 μM MG132. **b**, **c** RARγ regulates E-cadherin at transcriptional level. qPCR analysis of the E-cadherin expression in RARγ siRNA-transduced MHCC-97H and QGY-7703 cells (b) or RARγ-transfected MHCC-97H cells (c). **d**, **e** BMS-345541 inhibits RARγ-driven E-cadherin reduction. qPCR (d) or immunoblotting (e) analysis of the E-cadherin expression in RARγ-transfected Huh-7 cells treated with vehicle or 10 μM BMS-345541. **f**, **g** TNFα promotes RARγ-driven E-cadherin reduction. Immunoblotting analysis of the levels of E-cadherin expression in RARγ-transfected Huh-7 cells (f) or RARγ siRNA-transduced MHCC-97H cells (g) treated with vehicle or 20 nM TNFα. Statistical significance was determined by a two-tailed, unpaired Student's *t*-test. **p < 0.01. ns, no significance

The next task was to address how RARγ regulates E-cadherin expression. Huh-7 cells were treated with MG132, an inhibitor of protease, and then E-cadherin protein and mRNA levels were examined. However, RARγ-induced E-cadherin reduction at protein and mRNA levels was not affected by MG132 (Fig. 4a), indicating the regulation of E-cadherin expression by RARγ may occur at the transcriptional level. Real-time PCR assays further confirmed that silencing RARγ in MHCC-97H cells markedly induced E-cadherin mRNA expression (Fig. 4b), while ectopic RARγ expression in MHCC-97H cells largely impaired E-cadherin mRNA expression in a dose-dependent manner (Fig. 4c).

Given the fact that RARγ, like other nuclear receptors, can act as a transcription factor to regulate expression of its target gene through binding to its DNA response elements [39, 40], it is possible that RARγ may bind to the promoter of E-cadherin to regulate its expression. However, after sequence analysis of the region of E-cadherin promoter between -2000 bp and -1 bp, we did not find a potential RARγ binding site within the region (data not shown), suggesting that RARγ-driven E-cadherin reduction might be mediated by other molecules or signaling pathways. Interestingly, in the process of exploring which molecular or signaling pathway was involved in RARγ-induced E-cadherin reduction, we were surprised to find that BMS-345541, an inhibitor of NF-κB signaling pathway, completely blocked RARγ-induced E-cadherin reduction both at mRNA levels (Fig. 4d) and at protein levels (Fig. 4e) in Huh-7 cells. This suggests that the activation of NF-κB signaling is required for RARγ-induced E-cadherin reduction. To further confirm this finding, we treated MHCC-97H with tumor necrosis factor α (TNFα), an agonist against the NF-κB signaling pathway, and found that TNFα significantly enhanced RARγ-induced E-cadherin reduction (Fig. 4f) and greatly impaired upregulation of E-cadherin by silencing RARγ (Fig. 4g). Together, these results demonstrate that NF-κB signaling is indispensable for the regulation of E-cadherin by RARγ.

### RARγ expression is negatively correlated with E-cadherin expression in HCC cell lines and clinical HCC samples

**Fig. 5:**
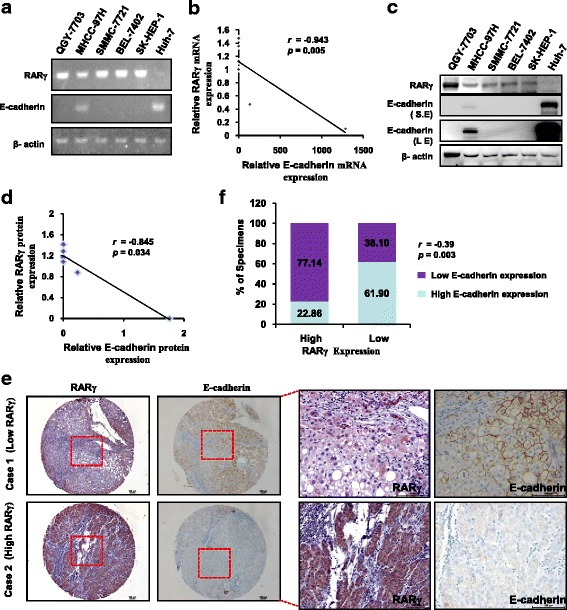
The expression levels of RARγ and E-cadherin in HCC cell lines and clinical HCC tissues. **a** The expression of RARγ and E-cadherin were evaluated by RT-PCR in the indicated cell lines. **b** Dot plot correlates the mRNA levels of RARγ and E-cadherin in HCC cell lines. The dotted line shows the negative correlation of RARγ and E-cadherin at the mRNA levels. **c** Immunoblotting analysis of RARγ and E-cadherin expression in the indicated cell lines. **d** The dot plot correlates RARγ and E-cadherin protein levels in six HCC cell lines. The dotted line shows the negative corralation of RARγ and E-cadherin at the protein levels. **e** Immunohistochemical staining of RARγ and E-cadherin in human CRC tissues. Representative bright-field images showing RARγ and E-cadherin staining in human HCC sections. Scale bar: 100 μm. **f** Spearman’s correlation analysis between RARγ and E-cadherin in 56 cases of HCC tissues

To further examine the RARγ-E-cadherin relationship, we analyzed the expression of RARγ and E-cadherin in HCC cell lines and clinical HCC samples. Interestingly, we notice a significant correlation in the expression of RARγ and E-cadherin both at the mRNA (Fig. 5a and b) and protein (Fig. 5c and d) levels in six HCC cell lines. The correlation of RARγ and E-cadherin was further validated by examining the expression of these two molecules in 56 cases of HCC tissues using immunohistochemical staining. Our results showed that low RARγ expression was associated with high E-cadherin expression in Case 1 (Fig. 5e). Inversely, the high levels of RARγ correlated with the low levels of E-cadherin in Case 2 (Fig. 5e). Spearman's Rank Correlation analysis confirmed that there is a significant negative correlation between RARγ and E-cadherin expression (Fig. 5f). Thus, these observations further strengthened our finding that RARγ promotes HCC invasion and metastasis through regulation of E-cadherin reduction.

## Discussion

Emerging evidence suggests that aberrant expression of nuclear receptor RARγ contributes to cancer development and progression [30, 31, 34]. In our previous studies, RARγ was identified as an oncogene that was associated with hepatocellular tumorigenesis by activating PI3K/Akt and NF-κB signaling pathway [31]. An overexpression of RARγ was found in HCC tissues, and its expression was closely correlated with the proliferation and growth of HCC cells [31], indicating its critical role in HCC development. However, it remains unclear whether aberrant expression of RARγ performs key roles in HCC invasion and metastasis.

In this study, we determined the significance and underlying mechanism for RARγ in HCC invasion and metastasis. The RARγ expression was markedly higher in HCC tissues with distant metastasis than in those HCC tissues without distant metastasis, suggesting a key role of RARγ in HCC progression. Analyzing the relationship between RARγ expression and pathological characteristics in 56 HCC patients by tissue microarray revealed a significant correlation of RARγ expression with TNM stages and distant metastasis. Kaplan-Meier analysis further showed that RARγ expression was closely correlated with the overall survival of HCC patients. Thus, these data indicated that RARγ might be considered a biomarker candidate for clinical HCC prognosis. The effect of RARγ on HCC cells invasion and metastasis was directly demonstrated in our in vitro and in vivo studies. Silencing RARγ significantly inhibits the invasive ability of HCC cells, and led to severe suppression of lung metastasis of HCC in mice. These observations are in good agreement with previous reports that high levels of RARγ expression in cholangiocarcinoma (CCA) promote CCA cell invasion [30].

Our current study demonstrated that RARγ acts as a metastasis-promoting protein in HCC through regulating NF-κB-dependent E-cadherin reduction. EMT plays a critical role in cancer metastasis. Cancer cells undergoing EMT acquire invasive properties [41]. Accumulating studies have revealed that EMT is a crucial mechanism in cancer progression and metastasis, and many proteins involve in this process [11]. In this study, we found that RARγ is indeed involved in EMT. Modulation of RARγ expression by siRNA or overexpression resulted in a significant change in E-cadherin expression. Loss or reduction of E-cadherin, an epithelium marker, is a hallmark of EMT. Loss or reduction of E-cadherin expression is often associated with the tumor grade and stage [42]. Several molecules and signaling have been identified to regulate E-cadherin expression by transcriptional or post-transcriptional mechanism [21, 25, 43]. We recently reported that upregulation of Nur77, an orphan member of the nuclear receptor superfamily, confers colorectal cancer invasive features through regulating MMP-9-dependent E-cadherin reduction [8]. One important finding reported here is that we identified inflammatory signaling NF-κB is involved in RARγ-driven EMT. Inhibition of NF-κB activity by pharmaceutical markedly impaired RARγ-induced E-cadherin reduction, while enhancement of NF-κB activity by inflammatory cytokine TNFα greatly promoted RARγ-induced E-cadherin reduction. These results suggest a requirement for inflammatory signaling NF-κB in RARγ-driven EMT. It is worthwhile to point out that recent studies revealed that NF-κB-mediated Snail stabilization might trigger inflammation-induced cancer cell migration and invasion [44]. This finding, together with ours, is in agreement with the notion that inflammatory tumor microenvironment facilitates both tumor development and metastatic progression. Indeed, epidemiologic studies have provided overwhelming evidence that chronic inflammation with hepatitis B virus (HBV) or hapatitis C virus (HCV) infections contributes to HCC development [6, 7], and extensive studies have revealed that genetically or chemically induced HCC depends on inflammatory signaling [45, 46]. Although we previously reported that RARγ-driven inflammatory signaling NF-κB promotes hepatocellular tumorigenesis [31], whether the mechanism also accounts for HCC invasion and metastasis is unknown. We here demonstrate that a key role of RARγ in induction of HCC metastasis through regulation of NF-κB-mediated E-cadherin downregulation.

## Conclusions

In summary, we have identified RARγ as a key regulator of HCC invasion and metastasis. RARγ upregulation in HCC cells and HCC tissues contributes to their proinvasive and prometastatic abilities in vitro and in vivo by regulating inflammatory signaling NF-κB-mediated E-cadherin reduction. This may highlight a new therapeutic opportunity for intervention of HCC metastasis by blocking RARγ-driven EMT.

## Additional files

Additional file 1: Figure S1. The efficiency of RARγ depletion in MHCC-97H. Immunoblotting of RARγ in MHCC-97H cells that stably transfected with shRNA/Control or shRNA/RARγ. (PDF 57 kb)
Additional file 2: Figure S2. Knockdown of RARγ does not affect the expression of Vimentin, N-cadherin and Occludin. qPCR analysis of the vimentin, N-cadherin and occludin expression in RARγ siRNA-transduced MHCC-97H cells. Statistical significance was determined by a two-tailed, unpaired Student's t-test. ns, no significance. (PDF 30 kb)

## Abbreviations

EMT: Epithelial-mesenchymal transition
HCC: Hepatocellular Carcinoma
qPCR: Quantitative PCR
