# Supplementary figures and images for: RARγ-induced E-cadherin downregulation promotes hepatocellular carcinoma invasion and metastasis
Source: J Exp Clin Cancer Res. 2016 Oct 19;35:164. doi: 10.1186/s13046-016-0441-9 (PMC5069892; doi:10.1186/s13046-016-0441-9)

# Supplementary Figure 1

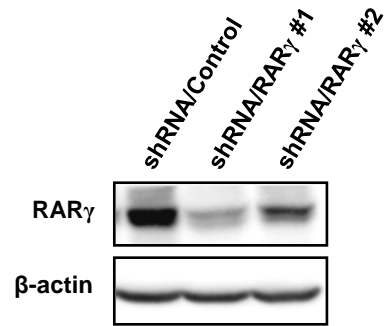

Supplement: Additional file 1: Figure S1. — The efficiency of RARγ depletion in MHCC-97H. Immunoblotting of RARγ in MHCC-97H cells that stably transfected with shRNA/Control or shRNA/RARγ. (PDF 57 kb) [file 13046_2016_441_MOESM1_ESM.pdf]

## Supplementary Figure 2

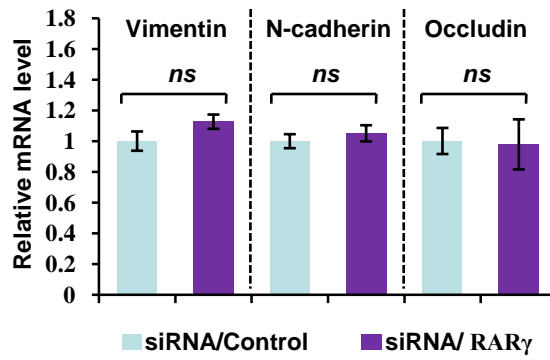

Supplement: Additional file 2: Figure S2. — Knockdown of RARγ does not affect the expression of Vimentin, N-cadherin and Occludin. qPCR analysis of the vimentin, N-cadherin and occludin expression in RARγ siRNA-transduced MHCC-97H cells. Statistical significance was determined by a two-tailed, unpaired Student's t-test. ns, no significance. (PDF 30 kb) [file 13046_2016_441_MOESM2_ESM.pdf]
